# Effects of Temperature Fluctuations on Cabernet Sauvignon Branches and Wine Grape Appellation Yields: An Analysis Based on the Standardized Temperature Adaptation Index

**DOI:** 10.3390/plants14121886

**Published:** 2025-06-19

**Authors:** Yunlong Ma, Jinyue Yang, Ping Wang, Guoli Cheng, Qinming Sun

**Affiliations:** 1 Agricultural College, Shihezi University, Shihezi 832003, China; 15225759926@163.com (Y.M.);; 2 Key Laboratory of Special Fruits & Vegetables Cultivation Physiology and Germplasm Resources Utilization of Xinjiang Production and Construction Corps., Shihezi 832003, China; 3 Food College, Shihezi University, Shihezi 832000, China; 4 Chateau Changyu Baron Balboa, Shihezi 832000, China

**Keywords:** climate change, clod stress, temperature variability, grapevine physiology, Cabernet

## Abstract

Climate fluctuations due to global warming significantly impact the wine grape industry. This study introduces the Standardized Temperature Adaptation Index (STAI), which is specifically designed to isolate temperature trends and quantify the effects of temperature fluctuations on low-temperature stress affecting Cabernet Sauvignon branches and yields in the wine grape production regions of Xinjiang. A low-temperature fluctuation experiment was conducted on Cabernet Sauvignon branches to simulate the temperature conditions and fluctuations experienced by wine grapes during the overwintering period. The treated branches then underwent recovery growth experiments, during which key physiological stress parameters were measured to assess the impact of temperature fluctuations on grape growth and development during overwintering. The results indicated that under identical low-temperature conditions, increased temperature fluctuations led to a 62% reduction in the budding rate of Cabernet Sauvignon branches, a 6% increase in relative conductivity, and elevated levels of proline, malondialdehyde, and soluble proteins. Additionally, the activities of superoxide dismutase, peroxidase, and catalase initially rose and then declined, indicating that temperature fluctuations intensified low-temperature stress. Data analysis from four wine grape production regions in Xinjiang between 2000 and 2020 revealed that temperature fluctuations corresponded with the peaks and troughs of yield fitting curves, demonstrating a negative correlation. As temperature fluctuations increased, yields decreased. The STAI introduced in this study is a straightforward, standardized measure that accurately reflects the effects of temperature fluctuations on grapes and is a valuable tool for future research on temperature variability and its impacts.

## 1. Introduction

Greenhouse gas emissions from anthropogenic activities have undoubtedly contributed to global warming. In its AR6 Synthesis Report: Climate Change 2023, the United Nations Intergovernmental Panel on Climate Change emphasized that climate volatility is rising in the context of global warming, leading to more frequent and intense climate extremes [1,2,3,4]. Climate change accounts for one-third of global crop yield variability, with climate volatility and extremes having an even more pronounced effect on yields than long-term climate trends [5,6,7]. The wine industry plays an important role in the global agricultural economy [8], and the climate substantially impacts wine grape quality more than soil conditions do [9]. Therefore, climate change can significantly affect wine production [10,11,12,13,14].

As global warming advances, the areas suitable for cultivating specific wine grape varieties are shifting dramatically [15,16]. With rising temperatures, suitable regions for wine grape cultivation are moving toward higher latitudes and altitudes [17,18]. For example, the suitable regions for Cabernet Sauvignon may shift from Bordeaux in France to higher-latitude areas, such as southern England and Germany, as well as to higher-altitude regions, such as the Andes and the Alps [19]. Temperature fluctuations due to climate change can reduce crop growth and productivity [20], as these fluctuations can trigger both beneficial and detrimental biochemical processes in plants [21], leading to a reduction in maximum light efficiency and CO_2_ fixation under the same light conditions [22]. However, other studies have demonstrated that controlled temperature fluctuations can enhance crop yield under specific conditions [23]. Given the ongoing global warming and the increase in temperature fluctuations, targeted analyses of how temperature fluctuations affect wine grape growth, development, and regional suitability are essential for supporting adaptations to climate change in the wine industry.

Numerous studies have investigated the characteristics of climate fluctuations and their impacts, particularly on precipitation. McKee et al. [24] introduced the Standardized Precipitation Index (SPI), a drought assessment tool derived from standardized precipitation data, which is widely used to characterize both the spatial and temporal variability of precipitation [25]. This index can detect drought characteristics across multiple scales and is frequently employed to analyze drought evolution and assess drought stress levels, as endorsed by the World Meteorological Organization [26,27,28]. For temperature, Zscheischler developed the Standardized Temperature Index (STI) based on the SPI and used it to analyze the spatial and temporal characteristics of temperature [29]. However, the STI was constructed using the expected value of temperature and thus does not accurately represent pure temperature fluctuations. In studies of temperature fluctuations, common methodologies include detrended fluctuation analysis, temperature distance levels, temperature variance analysis, and Fourier analysis [1,30,31,32]; however, these methods often struggle to represent simple temperature fluctuations adequately. Temperature fluctuations, even under the same average temperature conditions, can impact plant growth [33], and temperature changes have a significant influence on the grape industry [11]. However, research on the impact of the climate on grape growth and development has predominantly focused on long-term temperature change trends, with comparatively less attention given to the role of temperature fluctuations [17,34]. An index that reflects pure temperature fluctuations is essential for effectively assessing the effects of temperature fluctuations on wine grapes in the context of global warming.

This study examined the relationship between temperature fluctuations and the growth and development of wine grapes. Using the STI as a basis, the Standardized Temperature Adaptation Index (STAI) was developed to effectively remove the influence of temperature expectations from the analysis. By applying the STAI, this study systematically analyzed the effects of low-temperature fluctuations on Cabernet Sauvignon branches and the impact of temperature fluctuations on grape yield in four wine-grape-growing regions in Xinjiang from 2000 to 2020, thereby analyzing the impact of temperature fluctuations on grapevine growth and development during both the dormancy and growing periods. This study presents a methodology for accurately characterizing temperature fluctuations and clarifying their effects on wine grapes, providing a valuable tool and theoretical foundation for addressing climate fluctuations associated with global warming. These findings offer scientific guidance for viticulturists in adapting vineyards to climate change and establish a basis for future research in related fields.

## 2. Materials and Methods

### 2.1. Overview of the Study Area

**Figure 1:**
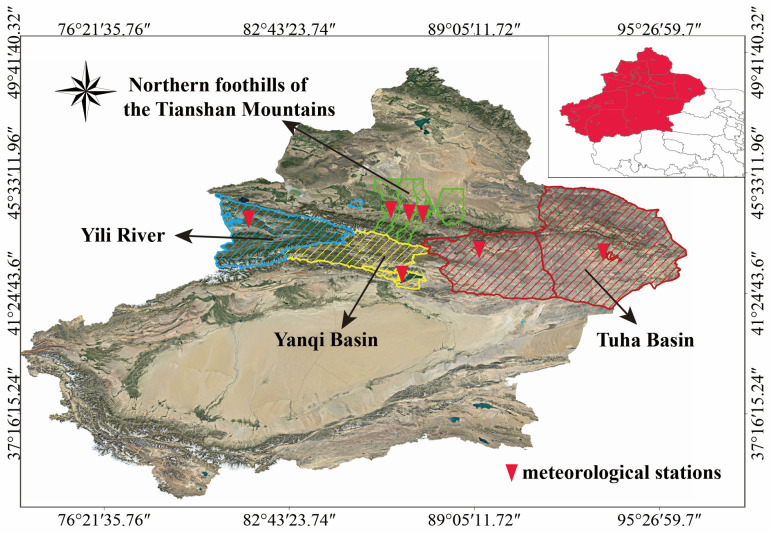
Overview of the study area. (color-coded: green for Northern foothills of the Tianshan Mountains, blue for Yili River Valley, yellow for Yanqi Basin, red for Tuha Basin).

The Xinjiang Uygur Autonomous Region is located in the prime grape-growing zone at 44° N latitude, characterized by extended sunshine hours and significantly elevated diurnal temperature variations. Over the past decade, Xinjiang has become China’s largest grape-producing area, with a cultivation area of 143,900 ha and an annual output of 270.57 million kg [35]. In 2023, the wine grape cultivation area in Xinjiang reached 20,000 ha, with an annual production capacity of 550,000 kiloliters for both grape wine and distilled spirits [36]. The primary production areas are concentrated in four regions (Figure 1): the northern foothills of the Tianshan Mountains, the Tuha Basin, the Yanqi Basin, and the Yili River Valley. The Tuha Basin, Yanqi Basin, and Yili River Valley production areas have frost-free periods of up to 180–220 d, with a high degree of dryness and low precipitation during the ripening period, which are conditions that support wine grape growth. The amount of precipitation during the growing and ripening seasons is ideal for wine grape development and promotes the accumulation of sugars and phenolics [37]. The production areas in the northern foothills of the Tianshan Mountains benefit from Tianshan Mountain snowmelt and groundwater. This region, which is rich in water resources and heat, supports the growth of wine grapes and is recognized as one of China’s premium wine grape production areas [38].

### 2.2. Data Sources

#### 2.2.1. Temperature Data During the Growing Season

**Figure 2:**
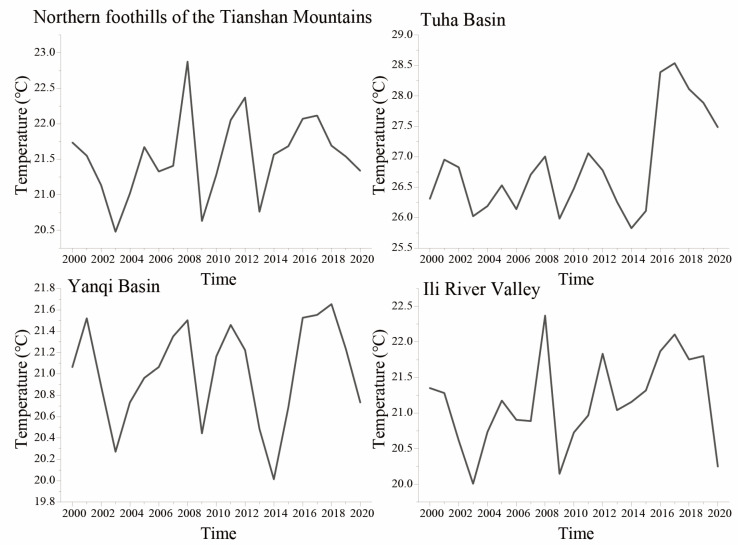
Annual growing season average temperature (2000–2020) in four production regions.

Meteorological data for the 2000–2020 growing seasons (May to September) in the four appellations were obtained from daily mean temperatures recorded at seven meteorological stations, with data for the northern foothills of the Tianshan Mountains sourced from the average values of three stations, data for the Tuha Basin sourced from two stations, and data for the Yanqi Basin and Ili River Valley sourced from a single station, all of which were selected based on their proximity to local vineyards cultivating wine grapes. Missing data accounted for less than 0.3% at all stations and were filled with data from a reference station using linear regression interpolation. Climate data were sourced from the National Climatic Data Center (www.ncei.noaa.gov, accessed on 12 October 2022). The annual growing-season average temperature in the four production regions from 2000 to 2020 is shown in Figure 2.

#### 2.2.2. Wine Grape Yield and Planting Area in the Production Area

Data on the production and planted areas from 2000 to 2020 were obtained from the Statistics Bureau of the Xinjiang Uygur Autonomous Region, the Statistics Bureau of the Xinjiang Production and Construction Corps, and enterprises within the production areas. The STAI was calculated using meteorological data from the four wine grape-producing regions in Xinjiang, and the unit area yield for each production region was calculated as the annual total wine grape production of that region divided by the planted area of that region. A fitting analysis was performed between the unit area yield data and the STAI over the 20-year period to evaluate the correlation between the two curves and assess the impact of temperature fluctuations on annual grape yields.

#### 2.2.3. Experiment on the Low-Temperature Fluctuations During the Wintering Period

Annual branches of Cabernet Sauvignon, grown under the same conditions as 5-year-old grapevines at the Baron Zhangyu Babao Winery in Shihezi City, Xinjiang—located in the northern foothills of the Tianshan Mountains within the study area—were selected for analysis, with a total of 140 branches being sampled. These branches exhibited consistent growth conditions, morphologies, and size, and they were collected six buds away from the base of each branch. The branches were cut into segments containing five bud nodes, rinsed under running water, washed two to three times with distilled water, wax-sealed at both ends to prevent water loss, and then sand-sealed at 4 °C for a duration of 40 d.

The sand-sealed branches were rinsed with distilled water and subsequently subjected to a low-temperature fluctuation treatment in an alternating incubator. This treatment consisted of seven different conditions, as outlined in Table 1. The CK group, which served as the control group in the experiment, was continuously treated at 4 °C for comparison with other treatment groups. For the cooling process, treatment groups B and E were cooled to the target temperature at a rate of 10 °C per second and maintained at that level for 24 h to simulate extreme climatic conditions. In contrast, treatment groups A, C, D, and F were gradually cooled to the target temperature at a rate of 4 °C per hour. Treatment groups A and D were held at the target temperature for 24 h, while treatment groups C and F underwent temperature fluctuations around the target temperature over 24 h to mimic long-term temperature variations. Treatment groups A, B, and C had an average temperature of −10 °C, with a cumulative negative temperature of −240 °C, whereas treatment groups D, E, and F had an average temperature of −20 °C, with a cumulative negative temperature of −480 °C. At the end of these treatments, 50 complete single-bud segments, each approximately 5 cm in length, were randomly selected from each replicate group for restoration growth testing. The remaining branches were sliced into thin sections (1–2 mm), excluding any bud nodes. The slices were thoroughly mixed and randomly sampled three times to measure the relevant indices.

Growth Recovery Experiment: Branches from the seven treatment groups were placed in a controlled environment chamber and maintained at a temperature of 25 °C, with a photoperiod of 16 h of light and 8 h of darkness and a humidity level of 70%. The branches were hydroponically cultivated for 20 d. Once the number of sprouted branches stabilized, the budburst rate was recorded to evaluate the recovery of growth potential across the different treatment groups.

The budburst rate was calculated using the following formula:

$$Budburst\;rate=\left(\frac{Number\;of\;sprouted\;branches}{Total\;number\;of\;branches\;in\;the\;treatment}\right)\times 100$$

Relative conductivity was determined following the method described by Zhang et al. [39]. The proline content was measured according to the Bates method [40]; malondialdehyde (MDA) levels were assessed using the thiobarbituric acid assay [41]; soluble protein content was evaluated with the Coomassie Brilliant Blue G-250 method; superoxide dismutase (SOD) activity was measured according to the protocol of Giannopolitis [42]; catalase (CAT) activity was determined using Sohn’s methodology [43]; and peroxidase (POD) activity was quantified following Hernandez’s approach [44]. These indicators were measured immediately after the treatment phase was completed.

STI and STAI values were computed separately for the cooling and treatment phases of each intervention. Subsequently, the impact of low-temperature fluctuations on Cabernet Sauvignon branches was assessed by integrating the STI and STAI values with the budburst rate and various physiological indices.

### 2.3. Construction of the STAI

In this study, the temperature variance per unit of time was selected to measure temperature fluctuations and variations to investigate their effects. Building upon the concepts utilized by McKee et al. in defining the SPI and the methodology employed by Zscheischler in defining the STI, this study proposes the STAI [24,29].

First, the standardized temperature variation index (STVI) was calculated using the same methodology as the SPI, as follows:

(1) A retrospective time series of temperature variations was obtained and fitted to a probability distribution.
(2) The estimated cumulative probability of current or retrospective temperature variations was derived from this probability distribution.
(3) The resulting cumulative probability was then transformed into a standard normal deviation with a mean of zero and a unit variance.
  (i) Standardized index
    (1) $$\begin{array}{c} {STI}_{j}=\frac{E_{j}\left(p\right)-A}{\sqrt{B}} \end{array}$$
    (2) $$\begin{array}{c} {STVI}_{j}=\frac{{var}_{j}\left(p\right)-C}{\sqrt{D}} \end{array}$$
    (3) $$\begin{array}{c} A=\frac{\sum_{j=1}^{n}E_{j}\left(p\right)}{n} \end{array}$$
    (4) $$\begin{array}{c} B=\frac{{\sum_{j=1}^{n}{(E}_{j}\left(p\right)-A)}^{2}}{n} \end{array}$$
    (5) $$\begin{array}{c} C=\frac{\sum_{j=1}^{n}{var}_{j}\left(p\right)}{n} \end{array}$$
    (6) $$\begin{array}{c} D=\frac{{\sum_{j=1}^{n}{(var}_{j}\left(p\right)-C)}^{2}}{n} \end{array}$$
  (ii) Here, *p* is the average temperature per unit of time (such as 1 month), *n* is the number of years, *E_j_*(*p*) is the average temperature per unit of time (5 months) in the *j*-*th* year, and *var_j_*(*p*) is the temperature variance per unit of time (5 months) in the *j*-*th* year.

Second, the influence of temperature variation on the STVI must be eliminated. The degree of temperature variation affects the temperature variance per unit of time, which is determined by both the average temperature and its fluctuations. Therefore, it is crucial to eliminate the impact of temperature variations. A correlation coefficient, ρ, exists between the STVI and STI, where ρ represents their causal relationship. An increase in temperature fluctuation does not change the average temperature, whereas a change in the average temperature leads to an increase in the fluctuations. Thus, ρ can be considered a causality coefficient that indicates how changes in average temperature affect fluctuations. To account for trends in changing temperatures, this study proposes a standardized measure called the STAI, which can be calculated using the following equation:

(7) $$\begin{array}{c} STAI=\frac{STVI}{\sqrt{{1-\rho }^{2}}}-\frac{\rho }{\sqrt{{1-\rho }^{2}}}STI \end{array}$$

The objective of this study was to comprehensively investigate the impact of climate change on grape physiology and yield in a specific region by analyzing trends in the STI and STAI. To achieve this aim, we employed the STI (X~N (0, 1)) and STVI (Y~N (0, 1)) as marginal distributions and fitted their joint distribution (STI, STVI)~N (0, 0, 1, 1, ρ) using a normal copula function construction, with ρ representing the correlation coefficient between STI and STAI. The following conclusions were drawn:

**Theorem 1.**  *When STI (X~N(0, 1)) and STVI (Y~N(0, 1)), Cov(X, Y) = ρ, and E(X^2^) = 1, and then the correlation coefficient between STAI (Z) and STI (X) is 0. The proof is provided in Appendix A.*

$$(1)Cov\left(\frac{Y}{\sqrt{1-\rho^{2}}}-\frac{\rho }{\sqrt{1-\rho^{2}}}X,X\right)=0$$

**Theorem 2.**  *The distribution* of $STAI=\frac{STVI}{\sqrt{{1-\rho }^{2}}}-\frac{\rho }{\sqrt{{1-\rho }^{2}}}STI$ *belongs to the standard normal distribution. The proof is provided in Appendix A.*

$$\left(2)STAI\left(Z=\frac{Y}{\sqrt{1-\rho^{2}}}-\frac{\rho X}{\sqrt{1-\rho^{2}}}\right)\sim N(0,1\right)$$

Therefore, the correlation coefficient between the STAI (Z) and STI was 0. In the joint distribution, (STI, STAI) follows a bivariate normal distribution, with a mean vector (0, 0) and covariance matrix [[1, 0], [0, 1]]. Notably, the STAI is independent of the STI. This characteristic renders the STAI as a suitable metric for evaluating temperature adaptation in response to fluctuations. Similar to other standardized indices, classification criteria can be applied to interpret STAI values: an STAI value within 1 standard deviation (−σ to σ) reflects normal conditions; values exceeding 2 standard deviations (STAI > 2σ) indicate extreme aggregation; and values below −2 standard deviations (STAI < −2σ) reflect extreme dispersion.

### 2.4. Analysis of Temperature Fluctuation Trends and Amplitude Sequences in Production Regions

The Mann−Kendall (MK) statistical test was used to analyze annual trends [45]. This study specifically focused on variations in the STAI and H_STAI_ across the four subregions by selecting daily average temperatures from May to September. To determine the maximum and minimum fluctuation periods *T =* [(*T*_1_ + *T*_2_) − 1] for a given sequence, X = {xi} (1 ≤ i ≤ n), *T*_1_ represents the length of the longest continuous increasing subsequence, and *T*_2_ denotes the length of the longest continuous decreasing subsequence. The amplitude of the periodic sequence was calculated. Consequently, this periodic amplitude sequence was defined as follows:

(8) $$\begin{array}{c} H_{x_{i}}={\max\left\{x_{j}\right\}}_{i-T\leq j\leq i}-{\min\left\{x_{j}\right\}}_{i-T\leq j\leq i} \end{array}$$

The series of cycle amplitudes can be utilized to analyze trends in the cycle amplitude, X = {x_i_}_(1 ≤ i ≤ n). Data from the grape growth period in the four major production regions were specifically chosen for the aforementioned calculation process and subjected to trend analysis.

### 2.5. Calculation of Comprehensive Stress Index

Principal Component Analysis (PCA) was performed on all the measured stress indicators and grapevine bud germination rate in this study. The first two principal components, which explain the highest cumulative variance, were selected. A weighted sum of these two principal components was calculated to obtain the Comprehensive Cold Stress Index for the assessment of adversity resistance.

(9) $$\begin{array}{c} S=\sum_{i=1}^{k}\omega_{i}\times P_{i} \end{array}$$

*S* is the comprehensive stress index, *ω_i_* is the weight of the i-th principal component, and *P_i_* is the score of the i-th principal component

### 2.6. Statistical Analysis

Data were statistically analyzed using IBM SPSS Statistics 23. Each treatment group was assessed with three replicates. An analysis of variance (ANOVA) was conducted to determine significant differences among the treatment groups. The results are expressed as means ± standard deviation (SD) of the three replicates. The significance of differences was marked using the letters a, b, and c, where different letters indicate statistically significant differences (*p* < 0.05) among the treatment groups. The letter annotations were applied separately for each indicator based on mean separations across the treatment groups.

## 3. Results and Analysis

### 3.1. Analysis of Low-Temperature Fluctuations in Cabernet Sauvignon Branches During Winter Dormancy Period

#### 3.1.1. Computation of the STI and STAI Under Varied Low-Temperature Fluctuation Treatment Conditions

**Figure 3:**
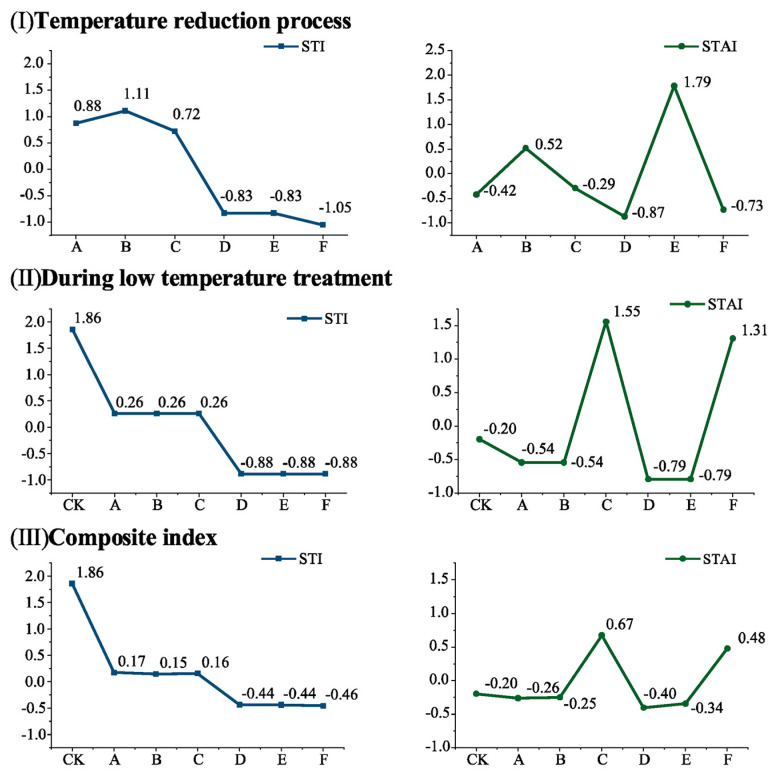
STI and STAI of Cabernet Sauvignon Branches during the low-temperature fluctuation treatments (CK: 4 °C, treated for 24 h. A: Gradually decrease to −10 °C at a rate of 4 °C/h, treated for 24 h; B: direct decrease to −10 °C, treated for 24 h; C: gradual decrease to −5 °C at a rate of 4 °C/h, alternately treated between −5 °C and −15 °C for 24 h; D: gradual decrease to −20 °C at a rate of 4 °C/h, treated for 24 h; E: direct decrease to −20 °C, treated for 24 h; F: gradual decrease to −15 °C at a rate of 4 °C/h, alternately treated between −15 °C and −25 °C for 24 h periods).

In the present study, to better capture the fluctuations of each treatment, three indices were computed for both the STI and STAI: (I) the cooling phase (CK treatment excluded in Fig. I due to absence of cooling process), (II) the low-temperature fluctuation treatment phase, and (III) the combination of (I) and (II) using time weights (Figure 3).

In (I), higher STAI values were obtained for treatments B and E than for the other treatments, indicating that the temperature fluctuation during the cooling process was the most severe in these treatments. In (II), similar STI values were obtained for treatments A, B, and C, as well as for treatments D, E, and F. Higher STAI values were observed for treatments C and F compared to the other treatments, suggesting that they experienced similar low-temperature conditions during the fluctuation process. However, the temperature fluctuations were more pronounced in treatments C and F than in the other treatments. In (III), treatment A had a similar STI value to treatment B but a lower STAI value; similarly, treatment D had a comparable STI value to treatment E but a lower STAI value. These results indicate that treatments A, B, and C shared similar low-temperature conditions throughout the experiment, with decreasing temperature fluctuations in the order of C > B > A. Similarly, for treatments D, E, and F, the low-temperature conditions exhibited a consistent pattern, with decreasing temperature fluctuations in the order of F > E > D. The calculated combination of the STI and STAI demonstrated a strong agreement with the observed low-temperature fluctuations in the experimental design of this study, indicating that the integrated utilization of the STI and STAI effectively captures the temperature dynamics and variations.

#### 3.1.2. Recovery Growth of Cabernet Sauvignon Shoots Under the Influence of Low-Temperature Fluctuations

**Figure 4:**
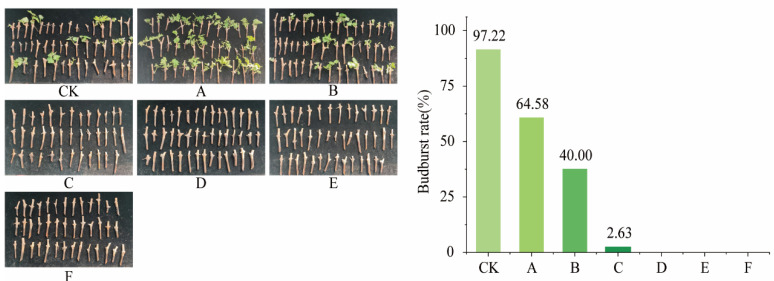
Budburst phenology of Cabernet Sauvignon branches following recovery from treatments simulating low-temperature fluctuations. (CK: 4 °C, treated for 24 h. A: Gradually decrease to −10 °C at a rate of 4 °C/h, treated for 24 h; B: direct decrease to −10 °C, treated for 24 h; C: gradual decrease to −5 °C at a rate of 4 °C/h, alternately treated between −5 °C and −15 °C for 24 h; D: gradual decrease to −20 °C at a rate of 4 °C/h, treated for 24 h; E: direct decrease to −20 °C, treated for 24 h; F: gradual decrease to −15 °C at a rate of 4 °C/h, alternately treated between −15 °C and −25 °C for 24 h periods).

The budburst rate of Cabernet Sauvignon branches was significantly lower after the low-temperature treatment than after the control treatment. Treatments D, E, and F, which had an average temperature of −20 °C, exhibited a budburst rate of 0% after recovery. Among the three treatments with an average temperature of −10 °C, treatment A exhibited a significantly higher budburst rate than treatment B, while treatment B had a significantly higher budburst rate than treatment C (Figure 4). Low temperatures had a significant impact on the budburst rate of Cabernet Sauvignon branches. Moreover, among the treatments with similar STI values, those with higher STAI values exhibited a more severe inhibition of Cabernet Sauvignon branch budburst. This suggests that under comparable temperature conditions, greater fluctuations in temperature have a more pronounced effect on Cabernet Sauvignon branch budburst and lead to a significant reduction in its overall rate.

#### 3.1.3. Repercussions of Low-Temperature Fluctuations on the Physiological Parameters of Cabernet Sauvignon Vines

**Figure 5:**
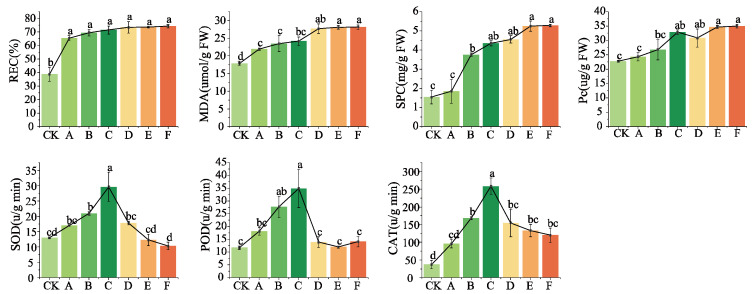
Alterations in the physiological indices of adversity in Cabernet Sauvignon branches following treatments involving fluctuations in low temperatures. Different superscript letters (a, b, c, d) indicate statistically significant differences (*p* < 0.05) among different treatments. (CK: 4 °C, treated for 24 h. A: Gradually decrease to −10 °C at a rate of 4 °C/h, treated for 24 h; B: direct decrease to −10 °C, treated for 24 h; C: gradual decrease to −5 °C at a rate of 4 °C/h, alternately treated between −5 °C and −15 °C for 24 h; D: gradual decrease to −20 °C at a rate of 4 °C/h, treated for 24 h; E: direct decrease to −20 °C, treated for 24 h; F: gradual decrease to −15 °C at a rate of 4 °C/h, alternately treated between −15 °C and −25 °C for 24 h periods; REC: relative conductivity; SPC: soluble protein content; MDA: malondialdehyde; Pc: proline content; SOD: superoxide dismutase; POD: peroxidase; CAT: catalase).

The results depicted in Figure 5 demonstrate that following the low-temperature fluctuation treatments, significant increases were observed in electrical conductivity, MDA content, soluble protein content, and proline (PRO) content across all treatment groups compared to the control group. Moreover, a clear positive correlation was observed between the severity of low-temperature fluctuations and increases in electrical conductivity, MDA content, soluble sugar content, and PRO content. Although significant differences in electrical conductivity were not observed among the treatments, notable variations were found in the MDA content between groups subjected to different average temperature treatments. Additionally, substantial disparities were detected in soluble protein and PRO content within the treatment groups at the same average temperature level.

The low-temperature fluctuation treatments increased the activities of the three antioxidant enzymes: SOD, POD, and CAT. In treatments A, B, and C, the activities of these three antioxidant enzymes exhibited an upward trend with the increase in STAI value. However, from treatment D onward, a decline in the activities of all three antioxidant enzymes was observed. This suggests that Cabernet Sauvignon branches were sensitive to temperature fluctuations, as indicated by their antioxidant enzyme activity. The reduction in antioxidant enzyme activity observed in treatments D, E, and F may be attributed to a certain level of low-temperature stress experienced by the branches, which led to the deactivation of these enzymes. The changes observed in the physiological indices of Cabernet Sauvignon branches indicated that as the treatment temperatures decreased further, the severity of low-temperature stress experienced by the branches intensified. Among the treatment groups with similar temperatures but varying degrees of fluctuation, an increase in the degree of fluctuation resulted in more severe low-temperature stress on the branches.

#### 3.1.4. Comprehensive Analysis of Low-Temperature Fluctuations’ Impact on Adversity Stress in Cabernet Sauvignon Branches

The budburst rate, PRO content, soluble protein content, relative conductivity, malondialdehyde content, and antioxidant enzyme activity assessed in this experiment were used to derive a composite index that reflects the adversity experienced by Cabernet Sauvignon branches through principal component analysis. Since the budburst rate for treatments D, E, and F was 0, only the four treatments, CK, A, B, and C, were considered when calculating the composite index. A higher value of the composite evaluation index indicated more severe stress on the Cabernet Sauvignon branches.

**Figure 6:**
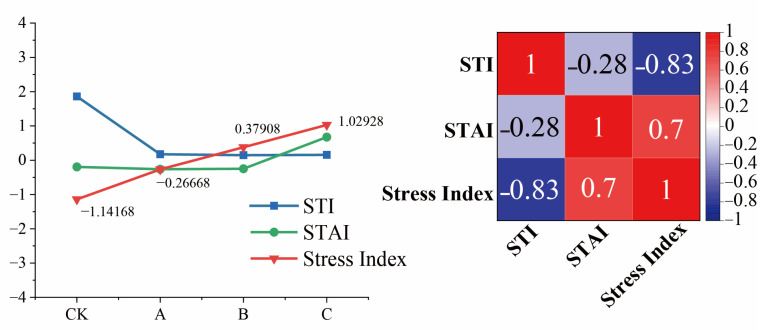
STI, STAI, and the Comprehensive Frost Injury Index of Cabernet Sauvignon branches following treatments involving fluctuations at low temperatures. (CK: 4 °C, treated for 24 h. A: Gradually decrease to −10 °C at a rate of 4 °C/h, treated for 24 h; B: direct decrease to −10 °C, treated for 24 h; C: gradual decrease to −5 °C at a rate of 4 °C/h, alternately treated between −5 °C and −15 °C for 24).

A correlation analysis among the knot stress index, STI, and STAI revealed that the stress index gradually increased from the CK to the C treatments. The STI and STAI values used in this analysis were derived from Section 3.1.1 of this study. The STI exhibited a decreasing trend, whereas the STAI showed an increasing pattern. Notably, a strong negative correlation was observed between the STI and the stress index, whereas a strong positive correlation was found between the STAI and the stress index (Figure 6). These findings suggest that as the temperature decreases, Cabernet Sauvignon branches experience an incremental increase in low-temperature stress. Furthermore, as temperature volatility increases, the stress endured by the Cabernet Sauvignon branches also rises.

### 3.2. Investigation of Temperature Fluctuations in Wine Grape Production Regions of Xinjiang

#### 3.2.1. Analysis of STAI Trends and Amplitude Sequences in Wine Grape Production Regions of Xinjiang

**Figure 7:**
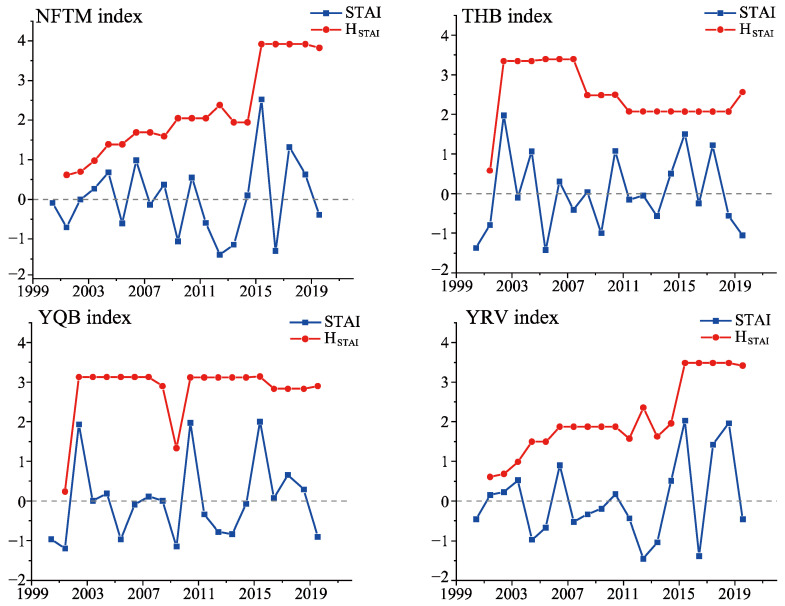
STAI and H_STAI_ sequences in four wine grape production regions in Xinjiang, China, from 2000 to 2020. NFTM = Northern Foothills of the Tianshan Mountains; HTB = Tuha Basin; YQB = Yanqi Basin; YRV = Yili River Valley.

The STAI values were calculated based on temperature data from the reproductive growth period of wine grapes across four regions over a 20-year period (with a span of 5 months) [46]. The results depicted in Figure 7 and Table 2 demonstrate that the slope of the STAI series during the wine grape-growing season in the four production areas ranged between >0 and <0.1, with a Z value below 1.96. This suggests an increasing trend in the STAI series throughout the growing season for wine grapes in these areas; however, this upward trend was not statistically significant. From 2000 to 2020, a weak upward trend in temperature fluctuations and a gradual decrease in temperature stability occurred during the growing season of wine grapes across all four production areas in Xinjiang.

The method of calculating cycle amplitude sequences was employed to compute the cycle amplitude sequences of the STAI, denoted as H_STAI_, in the four production regions. The 20-year H_STAI_ series for the four Xinjiang wine grape production areas revealed an upward trend in the northern foothills of the Tianshan Mountains and the Yili River Valley, a downward trend in the Tuha Basin, and a stable trend in the Yanqi Basin. The MK test at a 95% confidence interval indicated a significant upward trend in the H_STAI_ series for both the northern Tianshan Mountains and the Yili River Valley but a significant downward trend in the Tuha Basin. Over the past two decades, the cycle amplitude for the STAI series has gradually increased during the wine grape-growing season in the northern Tianshan Mountains and the Yili River Valley but has gradually decreased in the Tuha Basin. These findings demonstrate that temperature fluctuations during the wine grape-growing season became increasingly volatile within the northern Tianshan Mountains and the Yili River Valley from 2000 to 2020. In contrast, the temperature fluctuations within the Tuha Basin gradually decreased and showed increasing stability over this period. Furthermore, the temperature fluctuations within the Yanqi Basin’s production area tended to stabilize.

#### 3.2.2. Relationship Between Wine Grape Yield and STAI in Xinjiang Production Regions

The average annual yield of wine grapes in the four sub-production areas of Xinjiang exhibited a consistent upward trend, which is primarily attributed to advancements and innovations in viticulture technology, as well as varietal enhancements [47]. Notably, this growth trend was independent of temperature fluctuations. Therefore, first-order differences in the annual yield data of wine grapes were employed, and the polynomial models were subsequently fitted separately using the STAI series. Following this procedure, the correlation between temperature fluctuations and yield was analyzed during the grape-growing season by examining their relationship with the STAI.

**Figure 8:**
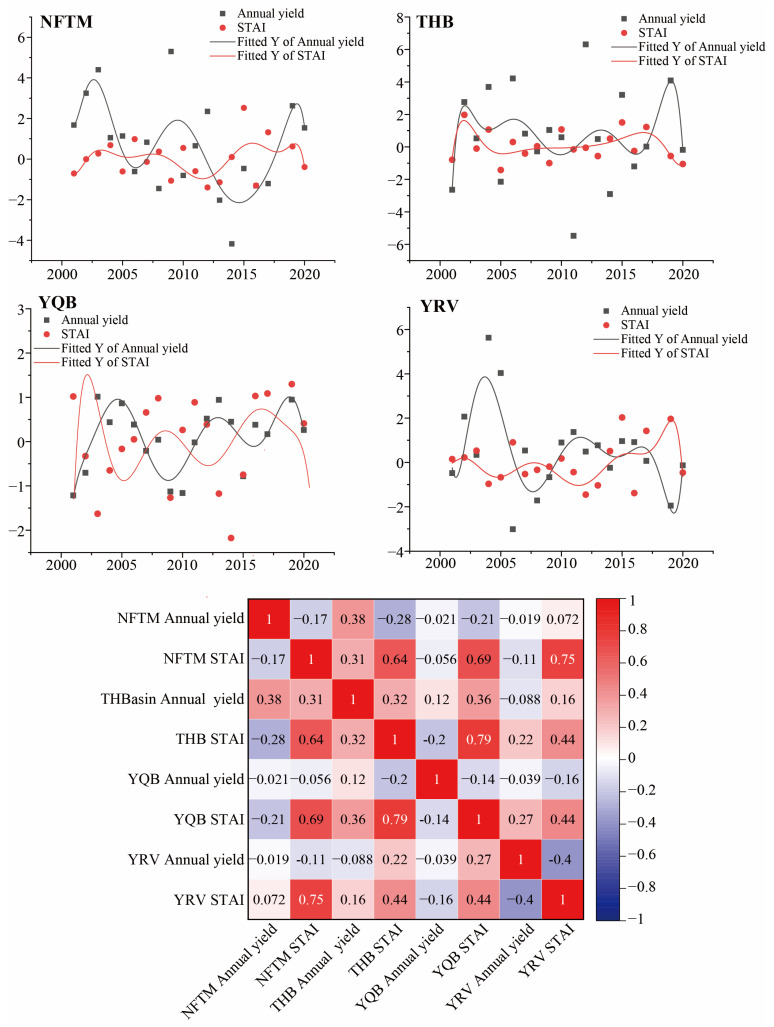
Annual yield and STAI index dynamics (2000–2020) in Xinjiang’s four major wine grape production regions: fitted trend curves (upper) and correlation heatmap (lower). NFTM = Northern Foothills of the Tianshan Mountains; HTB = Tuha Basin; YQB = Yanqi Basin; YRV = Yili River Valley.

The correlation coefficients between the growing season STAI series and the annual yield series of the four production areas—namely, the northern foothills of Tianshan Mountain, Tuha Basin, Yanqi Basin, and Yili Valley—were −0.17, 0.32, −0.14, and −0.4, respectively, for the period of 2000–2020 (Figure 8). The varying correlations between STAI and wine grape yield across different regions may be attributed to the distinct geographical locations and topographical features of these regions. The four regions encompass diverse terrains, including basins, valleys, and slopes, which result in different climatic conditions and microenvironments. These differences in climate and microenvironment influence the impact of temperature fluctuations on wine grape yield. The fitted curves revealed significant negative correlations between annual yields in each production area and the STAI, indicating the pronounced influence of temperature fluctuations on crop productivity. Notably, these fitted curves consistently demonstrated an inverse relationship between annual yields and the STAI in most years. Thus, as the STAI increased, the annual yield tended to decrease, and vice versa. This relationship highlights the detrimental impact of temperature fluctuations on wine grape output. In the production area of the northern foothills of the Tianshan Mountains, production increased when the STAI decreased between 2005 and 2015. Conversely, a decrease in production was observed when the STAI increased, highlighting the inhibitory effect of the STAI on yield. In the Tuha Basin production area, although the negative correlation between the STAI and yield curves was not pronounced, a declining trend in yield was observed as the STAI increased from 2005 to 2015. Significant inverse changes were observed in both the Yanqi Basin and Yili Valley production areas, particularly between 2002 and 2017. The peaks and troughs of both curves were consistent. Temperatures exceeding 24 °C during the growing season are considered unfavorable for grape growth [48]. In the Tuha Basin appellation, the percentage of days with average daily temperatures above 24 °C reached 74% between 2000 and 2020, significantly surpassing the range of 22% to 32% observed in the other three appellations. Considering the fitted curves, this indicates that temperature fluctuations have a more pronounced impact on yield within the suitable temperature range during the growing season, while their effect weakens at ambient temperatures that are unsuitable for grape growth.

## 4. Discussion

In this study, we propose that the STAI represents temperature fluctuations independent of the temperature trend by eliminating the influence of such trends from the STI. The STI was initially defined by Zscheischler based on the SPI to analyze the temporal and spatial characteristics of temperature [29]. The STAI is an extension of the STI, and both follow a standard normal distribution. Combining the STI and STAI allows for a more comprehensive expression of the relationships among temperature change, temperature fluctuations, and plant growth and development. Previous studies on this relationship have primarily focused on temperature trends and neglected the impact of temperature fluctuations on plant growth and development [49,50,51,52]. Under the influence of global warming, climate change not only impacts average temperatures but also affects temperature fluctuations [53]. In this study, we propose the STAI as a simple and direct method to investigate the relationship between temperature fluctuations and plant growth and development. The standardized deviation of the temperature variance was calculated based on the STAI to derive the STVI. This calculation allows for the comparison of temperature variances at different time points on a standardized scale, thereby enabling a clear visualization of momentary temperature fluctuations. However, since expectations can influence variance [54], we eliminated the influence of expected values by incorporating correlation coefficients into the calculation of the STAI. The STAI effectively expresses temperature fluctuations by focusing on variance while eliminating the influence of expected values.

The experiments on low-temperature fluctuations in Cabernet Sauvignon shoots demonstrated a significant decrease in the budburst rate as temperatures decreased. Furthermore, increasing temperature fluctuations resulted in further reductions in sprouting rates. Jun et al. showed that low temperatures can lead to the freezing of buds and surrounding tissues, thereby affecting grapevine shoot sprouting rates [55]. Under similar low-temperature conditions, temperature fluctuations can cause severe damage to grapevine shoot buds. In Cabernet Sauvignon, relative conductivity, MDA content, soluble proteins, and PRO content increased with low temperatures and temperature fluctuations. Ma et al. found that exposure to low temperatures led to an increase in relative conductivity and MDA content due to cell membrane damage in grapevine shoots, whereas Kishor et al. reported an increase in the concentration of soluble proteins and osmoregulators, such as PRO, following exposure to low temperatures [56,57]. Under low-temperature stress conditions, MDA and proline (PRO) are the most reliable physiological markers. MDA reflects lipid peroxidation damage, providing a direct assessment of cell membrane damage, making it a reliable indicator of oxidative stress; proline, on the other hand, helps plants cope with low temperatures by regulating osmotic pressure and stabilizing cell structures, thus serving as a key marker of cold tolerance. The degree of damage to the cell membrane system of Cabernet Sauvignon branches increased as both low temperatures and temperature fluctuations intensified. The activities of the three antioxidant enzymes—SOD, POD, and CAT—initially increased, followed by a decrease in response to low-temperature and temperature fluctuation stress in Cabernet Sauvignon branches. This suggests that Cabernet Sauvignon branches enhance the activity of antioxidant enzymes to scavenge reactive oxygen species (ROS) that accumulated after exposure to low-temperature stress [58]. However, as the severity of freezing stress in the branches increased, oxidative stress effects emerged, leading to damage to the corresponding enzyme systems and metabolic activities within the cells. Severe frost stress resulted in oxidative stress, causing further damage to these systems and decreased antioxidant enzyme activity [59].

This study revealed that as temperature fluctuations intensified under the same low-temperature conditions, the adversity stress indices of grapevine branches exhibited an upward trend. This suggests that increased temperature volatility leads to heightened low-temperature adversity stress in Cabernet Sauvignon branches in identical temperature scenarios. These findings align with the assertion of Aslam et al. that plants exhibit more intense biochemical responses under pronounced temperature fluctuation conditions than under stable conditions [21,60]. This study reveals that for wine grapes under cold stress conditions, both decreased temperatures and increased temperature fluctuations can intensify the low-temperature stress experienced by grapevines. Consequently, the proposed index offers a novel approach for investigating plant responses to low-temperature stress from the perspective of temperature fluctuations. Understanding the mechanisms that underlie low-temperature signal perception in plants is crucial for exploring their responses to low-temperature stress. Previous studies have demonstrated that the transmission of low-temperature signals primarily depends on cell membrane fluidity and calcium channels [61,62]. Additionally, plants synthesize various protective substances upon sensing low-temperature signals to regulate cellular osmotic pressure, mitigate reactive oxygen species accumulation, and impede extracellular ice nucleation [63], thereby enhancing their ability to withstand low-temperature stress. This phenomenon is referred to as the plant’s response to cold acclimation and is known as cold domestication [64,65]. Gradual cooling facilitates complete cold domestication in plants, enabling them to cope with severe low-temperature stress. Conversely, extreme cooling and temperature fluctuations may disrupt this process and expose plants to increased susceptibility to severe low-temperature stress [60].

In this study, by analyzing the STAI and its amplitude series in the Xinjiang production area, we found a weak upward trend in temperature volatility across the four sub-production areas during the period from 2000 to 2020, along with a significant increase in the volatility of temperature fluctuations in two of the production areas. The temperature in Xinjiang has exhibited an increasing trend over the past 50 years, and this warming trend is expected to continue, leading to an unstable climate state [66]. Climate fluctuations can affect both the yield and quality of wine grapes, as well as the varieties of wine produced [13,67]. In this study, we investigated the correlation between temperature volatility and wine grape yield and found that temperature volatility and yield during the growing season of wine grapes showed a negative correlation in three of the production areas but a weak positive correlation in the Tuha Basin. However, the fitted curves of STAI and yield in the four production areas exhibited contrasting characteristics, indicating a significant negative impact of temperature volatility on yield. This can be attributed to the high sensitivity of plants to temperature during the pollination stage, in which temperature changes can affect pollination rates [68]. Climate fluctuations throughout the growing season also led to variations in crop yield [69]. These findings highlight that increased temperature fluctuations can result in decreased wine grape yields, which is important for the planning and management of the wine grape industry. Therefore, maintaining stable temperatures within the growth environment is beneficial for the optimal growth and development of grape plants. During the reproductive growth period of wine grapes in viticultural regions, measures such as the use of shade nets, wind turbines, and irrigators can be employed to reduce temperature fluctuations, thereby helping to maintain stable yields.

The applicability of the STAI extends beyond its use in this study to investigate the impact of temperature fluctuations on plant physiological status and yield. Further comprehensive research on temperature and its variations can facilitate the planning and implementation of effective mitigation strategies in regions that are susceptible to temperature stress, thereby addressing the need for crops to enhance their adaptability to climate change [70]. In this study, we exclusively investigated the effect of temperature fluctuations on the growth and development of Cabernet Sauvignon varieties in the Xinjiang wine grape production area without considering the interaction between other climatic factors and temperature fluctuations. Therefore, it is important to acknowledge that the findings of this study have certain limitations. Future research should aim to further explore the combined impact of temperature fluctuations and other meteorological factors on different wine grape varieties under diverse climatic conditions to enhance our understanding of the STAI’s generalizability. The escalating influence of temperature fluctuations on agricultural production in the context of global warming necessitates effective analysis using standardized indices, which are valuable for evaluating variations in light, precipitation, wind, and other meteorological indicators.

## 5. Conclusions

This study proposes the STAI as a novel metric for characterizing temperature fluctuations and enabling a more accurate assessment of their impact on wine grapes by effectively mitigating the influence of temperature expectations on the manifestation of temperature volatility based on temperature variance. Compared to other temperature volatility indices, the STAI is not influenced by the mean temperature, allowing it to purely reflect temperature fluctuations. An investigation into the effects of low-temperature fluctuations on Cabernet Sauvignon branches revealed that decreasing temperature levels gradually intensified the cold stress experienced by these branches, whereas increasing temperature volatility at equivalent temperatures exacerbated this stress. Based on the analysis of temperature fluctuation characteristics in four major wine grape-producing regions in Xinjiang, China (i.e., the northern foothills of the Tianshan Mountains, Yanqi Basin, Yili Valley, and Tuha Basin), from 2000 to 2020, this study revealed that (a) temperature volatility has been increasing in the northern foothills of the Tianshan Mountains and both production areas of the Yili Valley; (b) temperature volatility tends to be stable in the Yanqi Basin producing regions; and (c) temperature volatility presents a decreasing trend in the Tuha Basin. Moreover, the stability of temperature fluctuations decreased in both production areas of the Yili Valley and increased in the Tuha Basin. Additionally, temperature fluctuations during the growing season negatively influenced grape yield, with a more pronounced impact within the optimal temperature range for grape cultivation. In unsuitable temperature ranges, the influence of temperature itself may become more significant, potentially reducing the impact of temperature fluctuations on yield. By incorporating novel indicators, such as the STAI, this study elucidated the mechanisms underlying the effects of temperature fluctuations on wine grape growth and productivity. Future research should aim to validate the applicability of the STAI across diverse regions and climatic conditions, investigate the interplay between temperature fluctuations and other environmental factors, and specifically examine the impact of temperature variations on different grape varieties. These efforts will contribute to enhancing the theoretical framework concerning temperature fluctuations and their influence on grape growth, thereby providing a scientific foundation for optimizing wine grape yield and quality while addressing climate change.

## Figures and Tables

**Table 1:** Details of the temperature treatments applied to the experimental samples.

Treatment Code	Treatment Details
CK	4 °C for 24 h
A	Decreased at a rate of 4 °C/h to −10 °C, treated for 24 h
B	Decreased at a rate of 10 °C/s to −10 °C, treated for 24 h
C	Decreased at a rate of 4 °C/h to −5 °C, alternately treated between −5 °C and −15 °C for 24 h
D	Decreased at a rate of 4 °C/h to −20 °C, treated for 24 h
E	Decreased at a rate of 10 °C/s to −20 °C, treated for 24 h
F	Decreased at a rate of 4 °C/h to −15 °C, alternately treated between −15 °C and −25 °C for 24 h

**Table 2:** Trend test results for the STAI and H_STAI_ in four wine grape production regions in Xinjiang, China (Z—standardized test statistic, b—Sen’s slope).

Region.	Index	Max	Mini	Z	b	Linear Slope
NFTM	STAI	2.52	−1.39	0.16	0.01	0.02
THB	STAI	1.97	−1.42	0.29	0.02	0.01
YQB	STAI	2.00	−1.19	0.94	0.02	0.02
YRV	STAI	2.03	−1.45	0.49	0.03	0.04
NFTM	H_STAI_	3.92	0.62	4.55	0.18	0.19
THB	H_STAI_	3.39	0.58	−2.49	−0.06	−0.05
YQB	H_STAI_	3.14	0.24	−1.54	0	0.03
YRV	H_STAI_	3.48	0.61	4.23	0.15	0.15

Footnote: NFTM = Northern Foothills of the Tianshan Mountains; HTB = Tuha Basin; YQB = Yanqi Basin; YRV = Yili River Valley.

## Data Availability

The original contributions presented in this study are included in the article. Further inquiries can be directed to the corresponding author.

## Appendix A

**Lemma A1.** *Let the two-dimensional joint density functions of (x, y) be p(x,y), if*$\left\{\begin{array}{c} u=g_{1}(x,y)\\ v=g_{2}(x,y) \end{array}\right.$*has continuous partial derivative and unique inverse function* $\left\{\begin{array}{c} x=x(u,v)\\ y=y(u,v) \end{array}\right.$*, $J=\left|\begin{array}{cc} \frac{\partial x}{\partial u} & \frac{\partial y}{\partial u}\\ \frac{\partial x}{\partial v} & \frac{\partial y}{\partial v} \end{array}\right|\neq 0$**. If $\left\{\begin{array}{c} U=g_{1}(X,Y)\\ V=g_{2}(X,Y) \end{array}\right.$**, then the two-dimensional joint density functions of (U, V) is p(u, v). $p\left(u,\;v\right)=p\left(x\left(u,\;v\right),y\left(u,\;v\right)\right)\left|J\right|$*

**Theorem A1.** 
*When STI (X~ N(0,1)) and STVI (Y~N (0,1)), Cov(X,Y) = ρ, E(X^2^)=1, then Cov (* $\frac{Y}{\sqrt{1-\rho^{2}}}-\frac{\rho }{\sqrt{1-\rho^{2}}}X$ *,X) = 0, namely the correlation coefficient between STAI (Z) and STI was 0.*

**Proof:** 

$SPAI(Z=\frac{Y}{\sqrt{1-\rho^{2}}}-\frac{\rho X}{\sqrt{1-\rho^{2}}})∼N(0,1)$

$Z=\frac{Y}{\sqrt{1-\rho^{2}}}-\frac{\rho X}{\sqrt{1-\rho^{2}}}$

$\;Cov\left(Z,X\right)=E\left(\left(\frac{Y}{\sqrt{1-\rho^{2}}}-\frac{\rho X}{\sqrt{1-\rho^{2}}}\right)X\right)-E\left(\frac{Y}{\sqrt{1-\rho^{2}}}-\frac{\rho X}{\sqrt{1-\rho^{2}}}\right)E\left(X\right)$

$=E\left(\frac{YX}{\sqrt{1-\rho^{2}}}-\frac{X^{2}\rho }{\sqrt{1-\rho^{2}}}\right)$

$=\frac{E\left(XY\right)}{\sqrt{1-\rho^{2}}}-\frac{\rho }{\sqrt{1-\rho^{2}}}E\left(X^{2}\right)$

$∵X∼N\left(0,1\right),∴E\left(X^{2}\right)=1$

$∵Cov\left(X,Y\right)=\rho$

∴E(XY)=ρ

□

**Theorem A2.** *The distribution of* $STAI=\frac{STVI}{\sqrt{1-\rho^{2}}}-\frac{\rho }{\sqrt{1-\rho^{2}}}STI$*belongs to the standard normal distribution.*

**Proof:** Let

$a=\frac{1}{\sqrt{1-\rho^{2}}},b=\frac{\rho }{\sqrt{1-\rho^{2}}}$

$\left\{\begin{array}{c} Z=aY-bX=U\\ X=V \end{array}\Rightarrow \left\{\begin{array}{c} Y=\frac{U+bV}{a}\\ X=V \end{array}\right.\right.$

cov(U,V)=0

$∴\frac{\partial x}{\partial u}=0,\frac{\partial y}{\partial u}=1/a,\frac{\partial x}{\partial v}=1,\frac{\partial y}{\partial v}=b/a$

$J=\left|\begin{array}{cc} \frac{\partial x}{\partial u} & \frac{\partial y}{\partial u}\\ \frac{\partial x}{\partial v} & \frac{\partial y}{\partial v} \end{array}\right|=\left|\begin{array}{cc} 0 & 1/a\\ 1 & b/a \end{array}\right|=-1/a$

$p\left(u,\;v\right)=p\left(x\left(u,\;v\right),y\left(u,\;v\right)\right)\left|J\right|$

$=\frac{1}{2\pi }\frac{1}{\sqrt{1-\rho^{2}}}\exp\{-\frac{1}{2\left(1-\rho^{2}\right)}(x^{2}-2\rho xy+y^{2})\}\sqrt{1-\rho^{2}}$

$=\frac{1}{2\pi }\exp\{-\frac{1}{2\left(1-\rho^{2}\right)}(x^{2}-2\rho xy+y^{2})\}$

$=\frac{1}{2\pi }\exp\{-\frac{1}{2\left(1-\rho^{2}\right)}(v^{2}-\frac{2\rho v\left(u+bv\right)}{a}+{\frac{\left(u+bv\right)}{a^{2}}}^{2})\}$

$P_{U}\left(u\right)=\int_{-\infty }^{+\infty }p\left(u,v\right)dv$

$=\int_{-\infty }^{+\infty }\frac{1}{2\pi }\exp\{-\frac{1}{2\left(1-\rho^{2}\right)}(v^{2}-\frac{2\rho v\left(u+bv\right)}{a}+{\frac{\left(u+bv\right)}{a^{2}}}^{2})\}dv$

$=\int_{-\infty }^{+\infty }\frac{1}{2\pi }\exp\{-\frac{1}{2}(v^{2}+u^{2})\}dv$

$=\frac{1}{2\pi }e^{-\frac{u^{2}}{2}}\int_{-\infty }^{+\infty }e^{-\frac{v^{2}}{2}}dv$

$=\frac{1}{\sqrt{2\pi }}e^{-\frac{u^{2}}{2}}$

$∴U∼N\left(0,1\right)$

$STAI(Z=\frac{Y}{\sqrt{1-\rho^{2}}}-\frac{\rho X}{\sqrt{1-\rho^{2}}})∼N(0,1)$

□
